# Supplementary material for: Comparing sentencing judgments of judges and laypeople: The role of justifications
Source: PLoS One. 2022 Nov 21;17(11):e0277939. doi: 10.1371/journal.pone.0277939 (PMC9678294; doi:10.1371/journal.pone.0277939)
Supplement: S1 File — (DOCX) [file pone.0277939.s001.docx]

**SI Vignette**

In February 2013, a then six-year-old boy died after being assaulted by his father. Since the spring of 2012, the father of the victim continued to severely assault the boy for extended periods every day, beating him with beer bottles, applying lit cigarettes to his body, and slamming him against the floor and wall. Furthermore, every night at 8:00 p.m., he would kick the boy out into the yard, lock him in a half-mat-sized steel storage room, and force him to sleep without bedding on the ground, stating that he would not allow him in the house until he became a good child. From December onward, the victim boy was unable to sleep at all in the warehouse due to freezing temperatures. He was extremely sleep-deprived and sometimes fell asleep crying even amid severe chastisement. In response to this, both parents would become angry, saying, “You have no remorse. You’re making fun of your parents.” Consequently, the assault escalated further. The boy’s only meal for the day was a loaf of bread, and when his mother forgot to buy it, he secretly ate dog food from the warehouse. When the boy complained that the dog food did not taste good, the defendant became furious, shouting, “Don’t be extravagant because you stole the food!” The boy sobbed and said, “Please forgive me. It’s delicious.” When the boy cried and apologized, the defendant allegedly continued to beat him. Furthermore, the victim had bronchial asthma but was not given any medication for it. Thus, he endured freezing temperatures and hunger every night and was not even allowed to drink water when he had asthma attacks. After ten months of severe assault and neglect, the child wasted away to less than half the average weight for his age, unable to stand or speak, or to go to the bathroom even when he wanted to with frequent incontinence. On the night before the child died of weakness, the accused abused the boy, who had almost lost consciousness, for more than seven hours by lecturing him severely, beating him with a beer bottle, slapping him, forcing him to sit on the floor, and threatening him with a kitchen knife. The accused finally dragged the child to a warehouse and confined him there. The boy was left in a sweatshirt over his underwear and bare feet. The temperature that day was minus 3 degrees Celsius. The next morning, the defendant found that the victim boy had died of weakness in the warehouse and buried his body in the mountains. The defendant said, “I was too strict in disciplining him. I didn’t mean to let him die.”
