# Supplementary material for: Comparing sentencing judgments of judges and laypeople: The role of justifications
Source: PLoS One. 2022 Nov 21;17(11):e0277939. doi: 10.1371/journal.pone.0277939 (PMC9678294; doi:10.1371/journal.pone.0277939)
Supplement: Attachment — Submitted filename: Response_to_Reviewers.docx [file pone.0277939.s002.docx]

**Response to Reviewers**

Thank you for reviewing our manuscript and giving us the opportunity to resubmit it. Please find in this letter a summary of the action we took to incorporate the points raised by you. The first part of this letter is our response to the additional requirements pointed out by the academic editor, and the second part is our response to the reviewer’s comments.

**Dear Editor,**

We apologize for not fully complying with PLOS ONE’s publication criteria. We thank you for indicating the details in this regard. We have thus, now incorporated the additional requirements into the revised version of the manuscript as mentioned below.

1. *Please ensure that your manuscript meets PLOS ONE’s style requirements, including those for file naming. The PLOS ONE style templates can be found at* 
[*https://journals.plos.org/plosone/s/file?id=wjVg/PLOSOne_formatting_sample_main_body.pdf*](https://journals.plos.org/plosone/s/file?id=wjVg/PLOSOne_formatting_sample_main_body.pdf) *and* [*https://journals.plos.org/plosone/s/file?id=ba62/PLOSOne_formatting_sample_title_authors_affiliations.pdf*](https://journals.plos.org/plosone/s/file?id=ba62/PLOSOne_formatting_sample_title_authors_affiliations.pdf)

Response 1: Thank you for your comment. We have ensured that the manuscript complies with the formatting samples that have been provided.

2. *Please provide additional details regarding participant consent. In the ethics statement in the* *Methods and online submission information, please ensure that you have specified what type you obtained (for instance, written or verbal, and if verbal, how it was documented and witnessed). If your study included minors, state whether you obtained consent from parents or guardians. If the need for consent was waived by the ethics committee, please include this information.*

Response 2: Thank you for your comment. We have now added information to the “Methods” section of the manuscript as well as the online submission information. The relevant section in the manuscript can be found at lines 115-118 on page 6.

3. *Please include your full ethics statement in the ‘Methods’ section of your manuscript file. In your statement, please include the full name of the IRB or ethics committee who approved or waived your study, as well as whether or not you obtained informed written or verbal consent. If consent was waived for your study, please include this information in your statement as well.*

Response 3: Thank you for your comment. Written informed consent was obtained electronically as is reflected in lines 127-129 on page 6.

**Dear Reviewer #1,**

We are extremely thankful to you for your helpful comments. Your comments provided valuable suggestions for improving the manuscript and furthered our research interests. The language, which received a negative response, has been now enhanced through a professional English editing service.

*This manuscript reports on an experiment in which lay participants sentencing decisions and justifications were compared with professional judges’ decisions in response to a child abuse case vignette. They conclude that judges are less motivated by retribution as a sole justification for punishment, and consequently they are more lenient. Overall, the study is interesting and well-justified. I have some comments that might help to polish the paper.*

Response: Thank you very much for your comment. We are glad that you appreciated our paper and we do appreciate your valuable comments.

*1. I don’t think your average reader will be familiar with the Kant/Bentham distinction so it would be beneficial to include a sentence or two defining retributive and utilitarian philosophies at the outset.*

Response 1: Thank you for your comment. As suggested, we have added the following few lines to the beginning of the Introduction:

This study examines “retributivism” versus “utilitarianism”—a jurisprudential issue regarding the justification of punishment—from the perspective of the differences between experts and non-experts. Retributivism implies that the essence of punishment is retribution for crime; and it seeks to offset crime through punishment. Utilitarianism is based on the idea that punishment should serve the function of preventing future crimes. It, therefore, seeks to deter recidivism and potential offenders from committing crimes.

*2. There are a few instances in the intro in which the sentences are a bit long and could be broken into multiple sentences for clarity.*

Response 2: Thank you for your comment. We have now reviewed the entire manuscript, including the introduction section, and revised the sentences according to your suggestion. In addition to shortening the sentences, we also revised the manuscript to simplify it by eliminating compound predicates.

*3. On p. 3 the word “capacitation” appears where I think the authors meant “incapacitation.”*

Response 3: Thank you for pointing it out. Our apologies. This was indeed our mistake. It has now been corrected (line 88 on page 4).

*4. Asking the participants for an open-ended justification seems like a clever strategy to encourage* *deeper processing of their decision. I do wonder if this promoted utilitarianism, given that it is considered the “cold calculated” decision (see Greene et al.’s 2001 dual process theory).*

Response 4: Thank you for your comment. As you may have guessed, the open-ended question was aimed to not only draw attention to the vignette but also served as a device to facilitate deeper processing. Your comment gave us an idea of what would happen if this device was not included or if the participants were under a high cognitive load, as in the criminal trial. Therefore, we have supplemented the manuscript with a discussion of this idea (lines 296-301 on page 13).

*5. I’m assuming the authors used a Bonferroni correction due to family-wise error concerns, but they don’t specify why or how they did this.*

Response 5: Thank you for your comment. The reason and method for the correction have been added to lines 194-196 on page 9: “To correct the family-wise error rate, multiple comparisons were conducted using the Bonferroni correction. The p-values were corrected in order of absolute t-values.”

6. *In the discussion, the authors note that because retribution was ranked third in importance that the measure was potentially inaccurate. However, the extant literature suggests that people tend toward utilitarian* *justifications while motivated by retribution (i.e., they like things like deterrence in theory but the underlying decision-making mechanism is actually retributive; see Carlsmith et al., 2002). For this reason, it’s interesting that the authors chose to include a scale that made the justifications* *mutually exclusive, forcing participants to choose. Lay people tend not to make the same distinctions that philosophers do, even in the face of logical incompatibility.*

Response 6: Thank you for your comment. We completely agree with the point made by you. The struggle between theory and motive is a major concern which motivated this study. We wanted to highlight their attitude of being rational in their emphasis on utilitarian justifications by also using a non-conflicting Likert scale in conjunction. However, the logic is that a conflicting ratio-type scale will force them to choose a genuine (accurate) response. We have thus added one sentence in the discussion section to clarify the same (lines 278-280 on page 12).

*7. Some details about the recruitment method are needed. Which company was used? Does this present any issues with selection bias? Unique demographics?*

Response 7: Thank you for your comment. We have added details about the recruitment process and a description of the Internet company (lines 113-115 on page 5) used for the survey: “Judges and laypeople were recruited from a panel of 2.2 million people representing the Japanese adult population (20 years and older) registered with the Internet survey company Rakuten Insight, Inc”. We assumed that the effect of selection bias was very weak because the company provided a sample that is representative of the Japanese population by using random sampling method. However, since the sample of judges was small, bias in age and gender was observed.

8. *Judges are notoriously difficult to recruit, which makes these findings a unique contribution. However, the reader needs more information about the characteristics of the judges - were they from the U.S.? How were they identified?*

Response 8: Thank you for your comment. Please see the first paragraph on page 5 (Materials and Methods section). We used an affiliate of one of the largest retail services in Japan (similar to Amazon). Judges who shop at that service are also registered. We recruited active judges from a very small sample of those registered as “highly qualified professionals.”

9. *How much were participants paid?*

Response 9: Thank you for your comment. They were compensated with shopping points worth US$0.2 (lines 124-126 on page 6).
