# Supplementary material for: Comparing sentencing judgments of judges and laypeople: The role of justifications
Source: PLoS One. 2022 Nov 21;17(11):e0277939. doi: 10.1371/journal.pone.0277939 (PMC9678294; doi:10.1371/journal.pone.0277939)
Supplement: Attachment — Submitted filename: Response to Reviewers.docx [file pone.0277939.s003.docx]

Dear Susan Yamamoto,

Editor

PLOS ONE

Thank you for reviewing our manuscript and giving us the opportunity to resubmit it.

We are very pleased to hear that you are satisfied with our previous response. Your insightful recommendations were in line with our intentions; therefore, we have revised the manuscript to reflect your suggestions.

*I served as Reviewer 1 in the initial submission and was invited to act as Guest Editor for the second submission. I was pleased with the thorough responses to my suggestions and believe each has been addressed appropriately. Per journal policy, a sole reviewer cannot serve as editor, and so a second reviewer’s feedback was requested. Reviewer 2 provides straightforward, minor recommendations, all of which I believe will enhance the final product and should therefore be addressed in the revision. I have some complementary recommendations which might assist in making these specific changes.*

*I agree with Reviewer 2’s second point that Kantian ethics are not well known to other professionals. Perhaps it would be helpful, for example on line 43, to simply say something like “Retributivism (which is based on Kant’s moral philosophy)…” then provide a source for a thorough review. Alternatively, you could remove reference to Kant and simply refer to something like “retributive focused philosophies”. Relatedly, for that same passage, I wonder if it’s more advantageous to use a word other than ‘retributive’ in its description, for example: “Retributivism implies that the essence of punishment is to provide just deserts for crime.”*

Response: Thank you for your valuable suggestions. We agree with your assessment that referencing Kant is unnecessary as Kantian ethics may not be well known. Hence, in the revised manuscript, we have deleted mentions of Kant.

*Regarding Reviewer 2’s comment about lines 162-167 on p. 7-8, this passage could indeed benefit from clarification. If I’m not mistaken, it expresses the idea that although punishment justifications are philosophically at odds and often incompatible in practice, Likert-type measures allow participants to rate high agreement with all of them. This strong endorsement of all justifications makes it difficult to identify the relative importance of each. Given your assertion that punishment is more likely a hybrid combination of motivations, in which some are prioritized over others, you forced participants to weight the relative importance via a scale summing 100. Is this interpretation correct? In any case, this passage should be reworded for clarity.*

Response: Thank you for understanding what we were trying to say. Your interpretation is perfectly correct. We have revised the passage as per your recommendations. (Page 8, Lines 165 to 168)

**Comments to the Author**

Reviewer #2: *In this interesting paper the authors compare layperson (i.e., nonexpert) and judge (i.e., expert) ratings of considerations for justice and sentence length in response to a vignette describing a fatal child abuse case. Most of my suggestions for the authors are stylistic and there are a few areas where I am unclear on what they are referring to:*

Response: Thank you for your comments. We apologize for any lack of clarity in sections of the manuscript. We have addressed all your comments as below; these revisions have greatly improved our manuscript.

*1. Some of the claims in the introduction need citations, such as the definitions of utilitarianism and retributivism, and the idea that media might be contributing to people's perceptions of crime, and the fact that people view child abuse as particularly heinous given the combination of lack of parenting duties fulfilled and the vulnerable victims.*

Response: Thank you for your comment. As per your suggestion, we have inserted citations where needed.

*2. Some of the concepts could benefit from more explanation. For example, I do not know what a "traditional Kantian moral code" is as someone who is 'adjacent' to your specific research area nor am I sure what "episodic media coverage" means*

Response: Thank you for your comment. We have changed “traditional Kantian moral code” to “retributive focused philosophies” throughout the manuscript in light of the editor's comment. (Page 3, Lines 49) According to Katz et al. (2019), child maltreatment is often taken up as an individual case that is reported on an episodic level rather than on a policy level as a social problem. (Page 4, Lines 83) Additionally, we have added necessary explanations for other concepts.

*3. On p. 5, line 95 the authors refer to judges as not being biased. I think it would be more accurate to say they are "trained to be less influenced by bias" because I'm sure some judges are indeed biased.*

Response: Thank you for your insightful comment. We have made the revision as you suggested. (Page 5, Line 97)

*4. On page 6 when the authors are describing the sample of judges, I am a bit confused - is the entire 50 person sample judges? On line 120-121 they also say "because there were too few judges, we aimed to recruit as many as possible and did not limit the demographic variables". I'm not sure if that means they recruited judges of any demographics or they recruited non-judges. I think just a simple edit of the sentence can make this more clear. Maybe something like "We permitted judges with any type of background demographics to ensure we could recruit 50."*

Response: As you surmised, all 50 participants were judges. We have made the correction as you suggested. (Page 6, Lines 122 to 124)

*5. p.7-8, likes 162-167 are a bit confusing, I am not entire sure what the authors are saying in these lines*

Response: Thank you for your comment. The editor has suggested that this section requires further clarification. The revised section is as follows: (Page 8, Lines 164 to 171).

However, while punishment justifications are philosophically at odds and often incompatible in practice, the Likert scale allows participants to rate high agreement with all of them (i.e., acquiescence-response; [35]). This strong endorsement of all justifications creates difficulties in identifying the relative importance of each [36]. Based on our theoretical definition that the psychological construct of sentencing is a hybrid of multiple justifications [37, 38], we deemed it more appropriate to examine the relative weighting of justifications on a ratio-type scale.

*6. I'm wondering why the authors decided to share the prosecutor's recommended sentence of 13 years in the sentencing decision vignette. Is it possible this influenced the responses? What was the reasoning for giving it? I apologize if I missed this detail in the paper.*

Response: Thank you for your important comments. In experiments where the severity of punishment is the dependent variable, the lack of a baseline, such as a plea, can lead to extreme responses and hinder the analysis (Watamura et al., 2014). Hence, we shared the plea; 13 years is a plausible plea in this vignette. We have added this explanation to the text to aid clarity. (Page 8, Lines 182 to 184)
